# Knowledge and behavior regarding pesticide use: a survey among caregivers of children aged 1–6 years from rural China

**DOI:** 10.1007/s11356-019-05560-w

**Published:** 2019-06-10

**Authors:** Yuanying Deng, Hongmei Dai, Ming Zeng, Lan Guan, Xiangwen Luo, Chen Zhang, Jing Tian, Jie Zhang, Ying Li, Qiong Xi, Mengwen Zhao, Mei Jiang, Lingling Zhao

**Affiliations:** 1 Department of Paediatrics, Third Xiangya Hospital of Central South University, Changsha, China; 2 School of Public Health, Central South University, Changsha, China; 3 Hunan Academy of Agricultural Sciences, Changsha, China; 4 Department of Neurology, Second Xiangya Hospital of Central South University, Changsha, China

**Keywords:** Pesticide exposure, Safety practices, Health, Left-behind children, China

## Abstract

Little is known about pesticide exposure risks for children in rural areas in China, many of whom have been left behind by migrant workers. To survey caregivers of children in rural China and assess their pesticide use and disposal methods, the measures were used to protect the children and their perceptions of the adverse effects of pesticides on children’s health. Three or four villages in each of Xinhua, Gongan and Sansui Counties in China were selected by random cluster sampling. The main caregivers of children aged 1–6 years were surveyed in face-to-face interviews. The questionnaire used was adapted from the World Health Organization “Exposure to Pesticides: Standard Protocol” survey and similar studies. The study included 464 caregivers (mean age, 46.4 years), who were most commonly the children’s grandparents (65.3%). Among the caregivers, 41.9% were educated to middle school level or higher, 45.4% had a household income < 297 USD, and 29.7% had received education/training about pesticide use/adverse health effects in children. The score for caregivers’ knowledge of the adverse effects of pesticides on children’s health was higher in those who had received education/training (5.1 ± 2.6 vs. 3.4 ± 2.5, *P* < 0.001). Factors associated with unsafe behaviors during pesticide use included grandparent as the caregiver (odds ratio [OR] 0.551; 95% confidence interval [95% CI] 0.368–0.824; *P* = 0.004), annual income < 297 USD (OR 0.580; 95% CI 0.395–0.853; *P* = 0.006), and insufficient health-related education/training (OR 0.436; 95% CI 0.286–0.665; *P* < 0.001). Improved education and training are needed to promote the safe use of pesticides by caregivers of children in rural China.

**Electronic supplementary material:**

The online version of this article (10.1007/s11356-019-05560-w) contains supplementary material, which is available to authorized users.

## Introduction

China is an agricultural country, and pesticides are widely used in agricultural production and daily life. China is now the world’s leading producer and consumer of pesticides and uses approximately 265,000 tons of pesticides for crops and plantations annually (Li et al. 2014). The use of pesticides in most regions of China lacks standardization, especially in rural areas, and limited knowledge and information regarding pesticide use among farmers in China likely contributes to some pesticides being overused (Li et al. 2014; Zhang et al. 2015). The utilization of pesticides is associated with deleterious health effects in farmers (Qiao et al. 2012; Zhang et al. 2016). Moreover, large-scale and nonstandard use of pesticides is associated with widespread health hazards due to the presence of pesticide residues in food, drinking water, soil, and air (Chung and Chen 2011; Crinnion 2009; Lai 2017; Yadav et al. 2015).

Children are more vulnerable than adults to environmental pesticides because they crawl and use eat in a hand-to-mouth manner (Ferguson et al. 2017). Furthermore, children have biological systems and organs that are still developing and are less able to eliminate environmental contaminants than are those of adults, making children more susceptible to disease following exposure to pesticides via skin contact, inhalation, or ingestion (Ferguson et al. 2017). Additionally, the risk of pesticide poisoning is increased when pesticides are not stored safely or securely, which may be a particular problem in rural regions (Jiang et al. 2010). Exposure to pesticides has been associated with many detrimental health effects that include acute toxicity (e.g., neurologic, gastrointestinal, ocular, respiratory, cutaneous, or cardiovascular) and chronic diseases such as cancer, birth defects, nervous system disorders, attention deficit hyperactivity disorder, and immune system abnormalities (Council On Environmental 2012; Lee et al. 2011; Roberts et al. 2012; Vidi et al. 2017) .

Children with less well-educated fathers, from lower income families and living in crowded conditions, are at greater risk of unintentional poisoning (ul Hassan et al. 2013). As most cases of childhood exposure to pesticides occur within the home, caregiver knowledge about the adverse effects of pesticides on children’s health and the safe storage of pesticides is an important factor in the prevention of unintentional exposure. However, the knowledge and correct use of pesticides are often inadequate, suggesting that educational interventions are merited to reduce the health risks of pesticide use (Nalwanga and Ssempebwa 2011). An additional issue particularly relevant to children in rural China is that many men have migrated to urban regions to seek higher wages, leaving behind their children, spouses and elderly parents (Yang 2013). In 2010, it was estimated that 58 million rural children were left behind in villages (Yang 2013). The 2010 national census revealed that the majority of rural left-behind children were located in major labor output provinces, and left-behind children accounted for more than 50% of all rural children in Hunan Province and more than 40% of those in Hebei and Guizhou Provinces. Left-behind children in rural China have been reported to have a reduced health-related quality of life (Jia et al. 2010) and a notably elevated incidence of farm-related injuries (Shen et al. 2013). However, there is little specific information about pesticide exposure risks for left-behind children in rural areas.

We hypothesized that children aged 1–6 years in rural areas might be at risk of pesticide exposure due to the absence of constant caregiver guidance. The aim of this study was to assess the caregivers’ methods of pesticide use and disposal in the home and farm, the measures used to protect children from exposure to pesticides, and the caregivers’ perceptions of the adverse effects of pesticides on children’s health. It was anticipated that this study would provide important information regarding pesticide usage in Chinese rural households with children aged 1–6 years.

## Methods

### Study participants

This study was a cluster survey of caregivers of children aged 1–6 years in three counties in China: Xinhua County in Hunan Province, Gongan County in Hubei Province, and Sansui County in Guizhou Province. These three counties were selected because they are regions that differ geographically: Xinhua County is mainly hilly, Gongan County is an area containing lakes, and Sansui County is a mountainous region. Agriculture is the main industry in all three counties, and different types of pesticides are used in these regions due to the diversity of crops (which include rice, cotton, and vegetables). We planned to select at least 200 children between 1 and 6 years for inclusion in our study. Based on the number of rural children in each county, three or four villages (4 villages in Xinhua County, 3 villages in Gongan County and 3 villages in Sansui County) in each county were selected by random cluster sampling. One of the main caregivers of each child aged 1–6 years in these villages was selected for participation in the survey.

The inclusion criteria were as follows: the caregiver was aged ≥ 18 years; the caregiver belonged to a farming family; the caregiver was able to understand the survey questions; and the caregiver provided informed consent for inclusion in the study. Only one caregiver from each family was eligible for the survey.

The study was conducted in accordance with the Declaration of Helsinki for the protection of human subjects and was approved by the ethics committee of the Third Xiangya Hospital of Central South University (Changsha, China). Prior to enrolment, all caregivers were given a verbal explanation of the research objectives, study procedures, and confidentiality of data handling. All caregivers provided informed written consent for inclusion in the study (a fingerprint was accepted instead of a signature when the caregiver was illiterate).

### Survey design and data collection

This study was a collaboration between the authors and the village doctors. Each village doctor asked all the caregivers in the village to take children aged 1–6 years to an assigned location for a free physical examination (during a specified period of time). The survey was administered at the time of the physical examination. Very few families did not take their children to the physical examination. In cases in which the family did not take their children to the physical examination, the authors visited the family in person (accompanied by a village cadre). Most caregivers were the grandparents of the children and were not able to read or write. If the caregiver was illiterate, the author and local doctor (or village cadre) explained each survey question to the caregiver, and the attending author was responsible for noting down the answer. The face-to-face interviews were conducted by trained professionals and took place between May 2013 and October 2013. Each interview took 30–40 min to complete.

The instrument used was a structured questionnaire that was developed based on a World Health Organization (WHO) field survey of pesticide exposure (Organization) and similar published studies. The questionnaire contained three sections. The first section collected demographic details such as age, sex, and educational levels. The second section contained questions to assess the caregiver’s behaviors and the measures used to protect children during the use of pesticides. The third section consisted of questions to assess the caregiver’s knowledge of the adverse effects of pesticides on children’s health. The specific questions asked and the scoring systems used for the second and third sections are detailed in the relevant tables.

### Statistical analysis

The raw data from the questionnaires were coded and entered into EpiData 3.1 software (www.EpiData.dk). The analysis was performed with SPSS 19.0 (IBM Corp., Armonk, NY, USA). Data are presented as *n* (%) or the mean ± standard deviation (SD). Data were tested for normality and compared between groups using Student’s *t* test or the chi-squared test, as appropriate. A *P* value < 0.05 was considered statistically significant. To determine which factors were associated with good behaviors regarding pesticide use, the caregivers were divided into two groups based on their behavior scores (total score ≤ 16 and total score > 16, based on the median total score), and univariate analysis was conducted using the chi-squared test. Then, variables with *P* < 0.05 in the univariate analysis were included in a multivariate logistic regression analysis to identify factors independently associated with a lower behavior score. Odds ratios (ORs) and 95% confidence intervals (95% CIs) were calculated for variables included in the multivariate analysis.

## Results

### Demographic characteristics of the caregivers

In total, 612 caregivers of rural children aged 1–6 years were surveyed, and 464 questionnaires were considered suitable for the analysis. The demographic characteristics of the caregivers included in the analysis are shown in Table 1. The mean age of the caregivers was 46.4 years, and most (60.7%) were aged ≥ 45 years. The majority of the participants were female (70.0%). The proportions of caregivers living in mountainous, lake, and hilly areas were 39.4%, 39.2%, and 21.3%, respectively. The majority of households (65.3%) had left-behind children, and only 34.7% of the caregivers were the parents of the children. Only 41.9% of the caregivers were educated to the middle school level or higher, and 45.4% of the caregivers had a household income less than 297 USD.

**Table 1:** Baseline demographic characteristics of the caregivers included in the study

	Number	Percentage
Gender		
Female	322	70.0%
Male	138	30.0%
Age (years)		
18–30	85	18.8%
31–44	93	20.5%
≥ 45	275	60.7%
Relationship with the child		
Parent	160	34.7%
Grandparent	301	65.3%
Education		
None/primary school	255	58.1%
Middle/high school	179	40.8%
College	5	1.1%
Annual income		
< 297 USD	208	45.4%
≥ 297 USD	250	54.6%
Region		
Hilly area	99	21.3%
Lake area	182	39.2%
Mountainous area	183	39.4%

Note that the total number of participants for each variable does not always add up to 464 due to missing data in some questionnaires

### Sources of information on pesticides

Most of the caregivers (50.0%) relied on relatives and friends for guidance on which pesticides to use. Point-of-sale merchants in shops selling farming supplies and technicians at the Department of Agriculture were also important sources of information and advice (40.8%). Only 9.2% of the caregivers relied on advertisements on the radio, television, and Internet for guidance on which pesticides to use. Only 42.5% of the respondents indicated that they read the manufacturer’s instructions before applying the pesticide. Some respondents believed that consulting the instructions was unnecessary because they already knew how to use the pesticide, while others did not read the instructions because they were illiterate. Only 29.7% of the respondents had received education regarding the adverse effects of pesticides on health and the correct use of pesticides.

### Caregivers’ knowledge of the adverse effects of pesticides on children’s health

Table 2 presents the survey findings regarding the caregivers’ knowledge of the adverse effects of pesticides on children’s health. Only 57.8% of all caregivers were aware that pesticides could enter the body through the skin, while 58.3% knew that pesticide exposure could lead to cancer in children, and 64.4% were aware that pesticide exposure could lead to hyperactivity in children. The vast majority of participants (98.8%) knew that pesticide poisoning necessitated a visit to the hospital, whereas very few caregivers were aware of other measures that needed to be taken after pesticide poisoning (Table 2).

**Table 2:** Caregivers’ knowledge about the adverse effects of pesticides on children’s health

Knowledge	Scoring system	All caregivers (*N* = 464)	Pesticide/health education (*N* = 138)	No pesticide/health education (*N* = 326)	*P*
Pesticides enter the body through the skin	Correct (2 pts)	259 (57.8%)	98 (71.5%)	91 (29.3%)	< 0.001
	Incorrect/unknown (0 pts)	189 (42.2%)	39 (28.5%)	220 (70.7%)	
Pesticide exposure leads to cancer in children	Correct (2 pts)	249 (58.3%)	73 (55.3%)	105 (35.6%)	< 0.001
	Incorrect/unknown (0 pts)	178 (41.7%)	59 (44.7%)	190 (64.4%)	
Pesticide exposure leads to hyperactivity in children	Correct (2 pts)	275 (64.4%)	68 (50.7%)	84 (28.7%)	< 0.001
	Incorrect/unknown (0 pts)	152 (35.6%)	66 (49.3%)	209 (71.3%)	
Measures that should be taken when pesticide poisoning occurs	Visit hospital (1 pt)	421 (98.8%)	131 (98.5%)	290 (99.0%)	0.670
	Take off clothes (1 pt)	47 (11.0%)	22 (16.5%)	25 (8.5%)	0.014
	Remove pesticide residue (1 pt)	49 (11.5%)	11 (8.3%)	38 (13.0%)	0.159
	Check pesticide name (1 pt)	29 (6.8%)	9 (6.8%)	20 (6.8%)	0.982
Total score	13	3.9 ± 2.7	5.1 ± 2.6	3.4 ± 2.5	< 0.001

Data are presented as *n* (%) or mean ± standard deviation. Note that the total number of participants for each variable does not always add up to 464 due to missing data in some questionnaires

The mean score for this section of the questionnaire was only 3.9 ± 2.7 (out of a maximum score of 13) and was significantly higher in caregivers who had received health education regarding pesticide use than in those who had not (5.1 ± 2.6 vs. 3.4 ± 2.5, *P* < 0.001). In addition, the scores in parents of the children were higher than those in grandparents (4.7 ± 2.8 vs. 3.5 ± 2.5, *P* < 0.001).

### Caregiver’s behaviors and measures used to protect children during the use of pesticides

Data regarding the caregivers’ behaviors regarding the use of pesticides and the measures used to protect children from exposure are shown in Table 3. Less than half of the participants (42.5%) read the label before using the pesticide. Most of the caregivers stored the pesticides (65.1%) and spraying tools (59.4%) in a high place that was out of reach of children (such as a high shelf), while over 10% stored these items randomly (Table 3). A minority of caregivers (9.5%) stored pesticides in other containers, but only 3.9% stored other items in pesticide containers. Notably, more than half of the participants (54.1%) did not use a professional recycling site or self-incineration for the disposal of pesticide waste or tools. Very few caregivers (7.3%) wore protective clothing during the application of pesticides, although most (82.3%) wore long-sleeved clothing. Furthermore, 21.4% of the respondents consumed food or drink or smoked cigarettes during the spraying of pesticides. The vast majority of caregivers surveyed washed their hands (94.2%) and took a bath (80.7%) immediately after pesticide application. However, less than half of the participants (46.7%) washed their clothing immediately after the application of pesticides, with 22.2% not washing their clothing at all following the use of pesticides. Furthermore, nearly a quarter of the caregivers (24.6%) did not wash the spraying tools following pesticide application. Only 0.4% of the caregivers surveyed set up a warning sign and provided oral information to prevent children from entering the farmland during the spraying or just after the spraying of pesticides, and 66.7% took no measures to prevent children from entering.

**Table 3:** Caregivers’ behaviors and measures used to protect children during the use of pesticides

Behavior	Scoring system	2 points	1 point	0 points
Read the label before pesticide application	Yes = 1; no = 0	–	197 (42.5%)	267 (57.5%)
Location of pesticide storage	High place = 2; storage room = 1; randomly placed = 0	299 (65.1%)	106 (23.1%)	54 (11.8%)
Location of pesticide-spraying tools	High place = 2; storage room = 1; randomly placed = 0	275 (59.4%)	138 (29.8%)	50 (10.8%)
Storing pesticides with other containers	Never did and thought you should not = 2; never did but thought you could = 1; yes = 0	389 (84.2%)	29 (6.3%)	44 (9.5%)
Storing other items in the pesticide container	Never did and thought you should not = 2; never did but thought you could = 1; yes = 0	412 (88.8%)	34 (7.3%)	18 (3.9%)
Disposal of pesticide waste or tools	Professional recycling site = 2; self incineration = 1; other = 0	99 (21.4%)	113 (24.5%)	250 (54.1%)
Eating, drinking or smoking during pesticide application	No = 1; yes = 0	–	364 (78.6%)	99 (21.4%)
Preventing children from entering farmland recently sprayed with pesticides	Oral information and warning sign erected = 2; oral information = 1; no measures = 0	2 (0.4%)	151 (32.8%)	307 (66.7%)
Washing hands following pesticide application	Yes and immediately = 2; yes but not immediately = 1; no = 0	435 (94.2%)	21 (4.5%)	6 (1.3%)
Taking a bath following pesticide application	Yes and immediately = 2; yes but not immediately = 1; no = 0	368 (80.7%)	64 (14.0%)	24 (5.3%)
Clothing worn during pesticide application	Protective clothing = 2; long sleeves = 1; short sleeves = 0	34 (7.3%)	381 (82.3%)	48 (10.4%)
Washing clothes used during pesticide application separately from children’s clothes	Yes = 2; occasionally = 1; no = 0	216 (46.7%)	144 (31.1%)	103 (22.2%)
Washing the spraying tools following pesticide application	Yes and immediately = 2; yes but not immediately = 1; no = 0	313 (67.5%)	37 (8.0%)	114 (24.6%)

Based on the scoring system detailed in Table 3, the average behavior score was 16.1 ± 2.9, and the score ranged from 7 to 23.

### Univariate and multivariate analyses of factors associated with good behaviors regarding pesticide use

Based on the behavior scores detailed in Table 3, the caregivers were divided into a “good behavior group” (total score > 16) and a “bad behavior group” (total score ≤ 16). Univariate analysis indicated that grandparent as the caregiver (vs. parent), annual income < 297 USD (vs. ≥ 297 USD), and education about pesticide use and its adverse health effects in children (vs. no education) were factors associated with unsafe behaviors (Table 4). Multivariate analysis (Table 4) revealed that the factors independently associated with unsafe behaviors regarding pesticide use included a grandparent as the caregiver (OR 0.551; 95% CI 0.368–0.824; *P* = 0.004), annual income < 297 USD (OR 0.580; 95% CI 0.395–0.853; *P* = 0.006), and insufficient health-related education/training in pesticide use (OR 0.436; 95% CI 0.286–0.665; *P* < 0.001).

**Table 4:** Univariate and multivariate analyses of factors associated with good behaviors regarding pesticide use

	Univariate analysis			Multivariate analysis*		
	OR	95% CI	*P*	OR	95% CI	*P*
Lake area (vs. mountainous area)	1.477	0.902–2.417	0.121			
Hilly area (vs. mountainous area)	0.656	0.401–1.075	0.094			
Grandparent as caregiver (vs. parent)	0.542	0.367–0.800	*0.002*	0.551	0.368–0.824	*0.004*
Age (+ 1 year)	0.991	0.979–1.004	0.176			
Education: illiterate/primary school (vs. middle school or higher)	0.944	0.652–1.366	0.760			
Annual income < 297 USD (vs. ≥ 297 USD)	0.633	0.437–0.917	*0.016*	0.580	0.395–0.853	*0.006*
Pesticide/health education (vs. no education)	0.455	0.302–0.686	*< 0.001*	0.436	0.286–0.665	*< 0.001*

*OR* odds ratio, *95% CI* 95% confidence interval

*Variables with *P* < 0.05 in the univariate analysis were entered into the multivariate analysis. Factors (Grandparent as caregiver, Annual income, Pesticide/health education) are associated with good behaviors regarding pesticide use (*P* < 0.05)

## Discussion

Notable findings of the present study were that approximately two-thirds of the households surveyed had left-behind children and that the child’s grandparent was the main caregiver in 65% of the cases. Less than half of the caregivers were educated to the middle-school level or higher, nearly half had a household income < 297 USD, and over 70% had not received education or training about pesticide use or the adverse health effects of pesticides in children. An important observation was that the score for caregivers’ knowledge about the adverse effects of pesticides on children’s health was significantly higher in participants who had received education/training in pesticide use or its adverse health effects than in participants who had not received this information. Furthermore, the factors associated with unsafe behaviors in pesticide use were a grandparent as the caregiver (vs. the parent), annual income < 297 USD (vs. income ≥ 297 USD), and insufficient health-related education/training. It is anticipated that the findings of this study will facilitate the development and implementation of effective strategies to increase awareness regarding the use of pesticides and the impacts of pesticides on children’s health and the environment. In addition, the findings may be useful for further studies of pesticide exposure and potential health effects in children.

It was evident from our research that, among the caregivers of the children, parents had more knowledge and exhibited better behaviors regarding the use of pesticides than grandparents. This is particularly relevant because the majority of households in our study (65.3%) had left-behind children. The grandparents of children make up the main farming workforce in rural China. Furthermore, the grandparents of children are usually from an older generation and have a relatively low education level, which limits their ability to provide effective care and guidance to the children (Shen et al. 2009). The main reason for acute pesticide poisoning in children is thought to be a lack of supervision, which results in the consumption of farming pesticides (Alavanja 2009). Moreover, the shortage among families of the labor needed for farmland maintenance makes it more likely that children will take part in farming activities, thereby increasing the risk of children being exposed to farming pesticides (Shen et al. 2009). Therefore, an effective way of reducing the exposure of children to pesticides is to increase their supervision. Cooperation between families, society, and schools could help provide children with more care and better supervision.

In the present study, most caregivers had never received education or training in the use of pesticides, and most relied on recommendations from their neighbors and relatives with regard to the selection of pesticides and methods of usage. Insufficient knowledge often leads to the misuse or overuse of pesticides and thereby to environmental pollution. A study in Uganda showed that friends and relatives had a contributory role in the choice of pesticides (Nalwanga and Ssempebwa 2011). An investigation in Egypt revealed that farmers who had received a school education had more knowledge of the negative effects of pesticides on health and the routes of contamination and higher scores on assessments of the precautions taken after exposure to pesticides (Ibitayo 2006). Another study also concluded that knowledge of the adverse effects of pesticides on health is an important factor affecting the safety measures adopted by farmers during the use of pesticides (Hashemi et al. 2012). Nonetheless, research in the Gaza strip indicated that the use of protective measures by farmers was poor, despite most farm workers being aware of the detrimental effects of pesticides on health (Yassin et al. 2002). Moreover, it has been reported that the provision of personal protective equipment improves the use of safety practices at work, while recent training was relevant to the use of safety practices at home (Strong et al. 2008). The WHO has estimated that there are more than 4 million acute unintentional pesticide-related illnesses and injuries annually, with up to 20,000 deaths related to unintentional pesticide poisoning (Levine and Doull 1992). Therefore, appropriate education and training would be an effective method of improving the knowledge and behaviors of caregivers regarding the use of pesticides. To achieve this, more media coverage (on television and in newspapers and magazines) is needed to emphasize the potential hazards of pesticides and the importance of using pesticides correctly. A simpler approach could be used for caregivers with a low level of education, such as sending them picture-based posters and diagrams that are easy to understand or arranging a visit from an agricultural technician who could explain the correct methods of selecting and using pesticides.

This study has some limitations. The caregivers included in the analysis were from only three counties in China, and the total number of participants was small in comparison to the number of farming households in China; thus, the generalizability of the findings is not known. The scoring rules for the questionnaire were not validated, so the results should be interpreted with a degree of caution. The number of cases of actual pesticide poisoning in the children and the factors associated with unintentional poisoning were not determined. The effects of educational and training interventions on pesticide use behaviors were not assessed. Further research is merited to extend our observations.

## Conclusion

Pesticides are considered a powerful tool for enhancing agricultural productivity in developing countries. However, pesticides are serious public health hazards to children due to incorrect usage and inadequate protection of children. Our data suggest that insufficient supervision of left-behind children by caregivers could increase the risk of pesticide exposure in children. Furthermore, a combination of factors contributes to unsafe behaviors in the use of pesticides by caregivers in rural China, and inadequate health-related education and training in pesticide use is an important factor. There is a need for the continuous provision of education and training in pesticide safety to the caregivers of children in rural China.

## Electronic supplementary material

ESM 1 (DOC 33 kb)

ESM 2 (DOC 42 kb)
