# Supplementary material for: Knowledge and behavior regarding pesticide use: a survey among caregivers of children aged 1–6 years from rural China
Source: Environ Sci Pollut Res Int. 2019 Jun 10;26(22):23037–43. doi: 10.1007/s11356-019-05560-w (PMC6658672; doi:10.1007/s11356-019-05560-w)
Supplement: Supplementary file 1 — (DOC 33 kb) [file 11356_2019_5560_MOESM1_ESM.doc]

**儿童编号： 儿童姓名： 出生日期： 年 月 日**

**儿童（1-6岁）生活环境与儿童健康问卷调查**

您好！为了了解1-6岁儿童的生活环境及健康状况，请您协助我们完成这次问卷调查并提供重要的反馈信息。希望您能认真、如实地填写，**在相应数字或□上打√**。感谢您的支持与合作！

| □儿童年龄为1-6岁 | □出生体重≥2.5kg | □37w≤出生时胎龄<40w |
| --- | --- | --- |
| □没有颅脑外伤史 | □没有严重的神经系统疾病史 |  |
| * 被调查者为孩子的主要照看人 | * 家里种田或小菜园 |  |

**联系电话：**

**家庭基本情况**

1. 孩子的主要照看人（可多选）：①父母 ②祖父母 ③外祖父母
2. 您的性别：①男 ②女 年龄： 岁
3. 您的文化程度：①小学及以下 ②初中 ③高中 ④大中专 ⑤大学及以上
4. 家庭人均年收入：①1000以下 ②1000~1500 ③1500~2000 ④3000以上
5. 家中有 亩田，主要种植农作物为（3种） 、 、 ；
6. 孩子的居住状态：①常住 ②暂住（一年居住时间少于6个月）
7. 孩子玩耍地点（可多选）：①农田/果园/大棚 ②家里及家附近 ③学校

**生活环境调查**

1. 种田者是怎样选择农药的（可多选）：①技术人员或销售人员指导 ②周围人推荐 ③网络、广播等广告宣传
2. 种田者购买农药时会仔细阅读标签吗？①会 ②不会
3. 家中存放农药的地点：①高处，不易被孩子拿到 ②杂物室 ③随意放置
4. 家中存放施药器具的地点：①高处，不易被孩子拿到 ②杂物室 ③随意放置
5. 可以用农药瓶以外的容器来保存农药吗？①可以，曾经用过 ②可以，但没有用过 ③不可以
6. 可以用用过的农药容器来存放其它物品（如食物、洗衣粉等）吗？①可以，曾经用过 ②可以，但没有用过 ③不可以
7. 通常如何处理废弃农药及其容器：①专业农药回收处 ②普通生活垃圾堆 ③随意丢弃
8. 种田者喷洒农药时或在刚用过药的农田是否有饮酒、吃东西、吸烟的行为：①是 ②否
9. 刚用过农药的农田是否会防止他人的立即进入？ ①不会注意 ②口头告知 ③口头告知并且立牌子等标记
10. 种田者在使用农药后会洗手吗？①会，立即洗手 ②会，但不是马上洗手 ③不会注意洗手的问题
11. 种田者在使用农药后回到家里会洗澡吗？①会，立即 ②会，但要过一段时间 ③不洗澡
12. 种田者在喷洒农药的时候会什么衣裤？①专业防护服 ②短袖衣、短裤 ③长袖衣、长裤
13. 种田者更换下来的衣服会与孩子的衣服分开清洗吗？ ①不会 ②偶尔 ③每次
14. 喷洒农药的器具使用后会清洗吗？①会，立即清洗 ②会，但要过一段时间 ③从来不洗

**儿童健康认知**

1. 您曾经接触过或者了解过关于“农药使用与儿童健康”的宣传吗？①是的 ②没有
2. 您认为皮肤接触农药是否可能会中毒？①会 ②不会 ③不清楚
3. 您认为孩子患癌症（如白血病、脑癌等）可能与接触农药有关系吗？①有关系 ②没有 ③不清楚
4. 您认为孩子注意力不集中、多动可能与接触农药有关系吗？①有关系 ②没有关系 ③不清楚
5. 如果发现孩子发生了农药中毒，您认为下列何种处理是必要的（可多选）：①急送医院 ②脱掉被污染的衣物、鞋、袜 ③清除口中的残留毒物 ④确定中毒农药名称

调查人员签字：

调查日期：
