# Supplementary material for: Knowledge and behavior regarding pesticide use: a survey among caregivers of children aged 1–6 years from rural China
Source: Environ Sci Pollut Res Int. 2019 Jun 10;26(22):23037–43. doi: 10.1007/s11356-019-05560-w (PMC6658672; doi:10.1007/s11356-019-05560-w)
Supplement: Supplementary file 2 — (DOC 42 kb) [file 11356_2019_5560_MOESM2_ESM.doc]

**No. Name: Birth date：**

Questionnaire Survey on Children's Living Environment and Children's Health

In order to understand the living environment and health status of children aged 1-6, please help us to complete the questionnaire and provide important feedback. I hope you can fill in the form truthfully, and type on the corresponding figures or**√**. Thank you for your support and cooperation!

| □age of children between 1-6 years | □37w≤gestational age<40w | | □birth weight ≥2.5kg |
| --- | --- | --- | --- |
| □No history of craniocerebral trauma | □No history of serious neurological disorders | | |
| □The respondents were the main caregivers for the children | | □farming family | |

**Tel：**

**Basic Conditions of family**

1. Children’s caregiver：①parents ②garndparents
2. Gender：①Male ②Female Age：
3. degree of education：①primary school and below ②Junior middle school ③high school ④Secondary Technical School ⑤College degree and above
4. average income of family/year：①<1000 ②1000~1500 ③1500~2000 ④>3000
5. acres of land in family，List three main crops in your family） 、 、 ；
6. How long do children live in the countryside every year：①>6 months ②<6 months
7. Places where children usually play (Multiple choices)：①farmland/orchard/egetable field ②indoors at home/ near home ③Kindergarten

**Surveys of caregiver’s behaviors of the use of pesticides**

1. How to choose pesticides(Multiple choices)：①relatives and friends’ guidance ②point of sale merchants in shops selling farming supplies/technicians at the department of agriculture ③advertisements on the radio, TV and internet
2. Whether read manufacturer’s instructions？① yes ②no why
3. Location of pesticide storage：①high place ②storage room ③randomly placed
4. Location of pesticide-spraying tools:①high place ②storage room ③randomly placed
5. Whether storing pesticides with other containers:①Never did and thought you should not ②never did but thought you could ③yes
6. Whether storing other items in the pesticide container:①Never did and thought you should not ②never did but thought you could ③yes
7. Disposal of pesticide waste or tools: ① Professional recycling site ② selfincineration ③ other
8. Eating, drinking or smoking during pesticide application：①yes ②no
9. Preventing children from entering farmland recently sprayed with pesticides？ ①Oral information and warning sign erected ②oral information ③no measures
10. Washing hands following pesticide application？①Yes and immediately ②yes but not immediately ③no
11. Taking a bath following pesticide application？①Yes and immediately ②yes but not immediately ③no
12. Clothing worn during pesticide application①Protective clothing ②long sleeves ③short sleeves
13. Washing clothes used during application separately from children’s clothes①yes ②occasionally ③no
14. Washing the spraying tools following pesticide application ①Yes and immediately ②yes but not immediately ③no

**Kowledge about effects of pesticides on children’s health**

1. Whether received education about the adverse effects of pesticides on health and about the correct use of pesticides? ① yes ②no
2. Pesticides enter the body through the skin ① yes ②no ③unknown
3. Pesticide exposure leads to cancer in children ① yes ②no ③unknown
4. Pesticide exposure leads to hyperactivity in children① yes ②no ③unknown
5. Measures that should be taken when pesticide poisoning occurs（Multiple choices）：① Visit hospital ② Take off clothes ③Remove pesticide residue ④Check pesticide name

Investigator：

Date：
